# Distinct Changes in Microbiota-Mediated Intestinal Metabolites and Immune Responses Induced by Different Antibiotics

**DOI:** 10.3390/antibiotics11121762

**Published:** 2022-12-06

**Authors:** Sunghyun Yoon, Giljae Lee, Junsun Yu, Kiuk Lee, Kyeongju Lee, Jiyeon Si, Hyun Ju You, GwangPyo Ko

**Affiliations:** 1 Department of Environmental Health Sciences, Graduate School of Public Health, Seoul National University, Seoul 08826, Republic of Korea; 2 Bio-MAX/N-Bio, Seoul National University, Seoul 08826, Republic of Korea; 3 KoBioLabs, Inc., Seoul 13488, Republic of Korea; 4 Institute of Health and Environment, Seoul National University, Seoul 08826, Republic of Korea; 5 Natural Products Research Center, Korea Institute of Science and Technology (KIST), Gangneung 25451, Republic of Korea; 6 Center for Human and Environmental Microbiome, Institute of Health and Environment, Seoul National University, Seoul 08826, Republic of Korea

**Keywords:** microbiome, intestinal metabolites, Th17 cells, ampicillin, vancomycin, *Parabacteroides*, *Akkermansia*

## Abstract

The cocktails of antibiotics are utilized to study the functions of microbiota. There have been studies on the alteration of not only the microbiota composition but also the host’s metabolism or immunity. However, the bacterial species associated with these altered physiologic markers are still unclear. Therefore, we supplied mice with drinking water containing ampicillin (AMP), vancomycin (VAN), neomycin (NEO), or metronidazole (MET) to observe the effect of each antibiotic on helper T cells and inflammation-related gene expression and metabolism, including amino acid metabolism and changes in gut microbiota. We observed major changes in gut microbiota in mice treated with AMP and VAN, respectively, immediately after administration. The abundance of the genera *Parabacteroides* and *Akkermansia* increased in the AMP and VAN groups, while *Prevotella* almost disappeared from both groups. The compositional changes in intestinal metabolites in the AMP and VAN groups were more distinct than those in the NEO and MET groups, which was similar to the microbiome results. In particular, the most distinct changes were observed in amino acid related metabolism in AMP and VAN groups; the amounts of phenylalanine and tyrosine were increased in the AMP group while those were decreased in the VAN group. The changed amounts of intestinal amino acids in each of the AMP and VAN groups were correlated with increases in the abundance of the genera *Parabacteroides* and *Akkermansia* in the AMP and VAN groups, respectively. The most distinctive changes in intestinal gene expression were observed in the ileum, especially the expression Th17-related genes such as *rorgt*, *il17a*, and *il17f*, which decreased dramatically in the guts of most of the antibiotic-treated groups. These changes were also associated with a significant decrease in *Prevotella* in both the AMP and VAN groups. Taken together, these findings indicate that changes in gut microbiota as well as host physiology, including host metabolism and immunity, differ depending on the types of antibiotics, and the antibiotic-induced gut microbiota alteration has a correlation with host physiology such as host metabolic or immunological status. Thus, the immune and metabolic status of the host should be taken into account when administering antibiotics.

## 1. Introduction

The gut is a major habitat for microbial communities residing in the human body; indeed, it is colonized by trillions of bacteria. Colonization of the gut is initiated immediately after birth. However, the composition of the bacteria is subject to alteration by species acquired from various environments. The intestine forms part of both the digestive and immune systems, because it is constantly exposed to antigens, including microbes. In response to these antigens, the gut has developed its own immune characteristics to maintain homeostasis. In particular, microorganisms play important roles in intestinal immunological functions. A previous study shows that germ-free mice have defective immunological function in the gut (including mesenteric lymph nodes (MLN) and Peyer’s patches) [1]. Studies in germ-free mice also show that Peyer’s patches are smaller, and that the number of CD4+ T cells and IgA-plasma cells is lower, in animals raised in the absence of microorganisms [2]. Moreover, germ-free mice show functional defects in T helper 17 (Th17) cells [3] and regulatory T (Treg) cells [4].

In addition to immunity, the gut microbiota impacts host metabolism [5]. Host metabolism comprises both microbial metabolism and the host’s own metabolism because the two are entwined [6]. The gut microbiota connect the intestine to other organs such as the liver and brain; this, in turn, regulates systemic metabolism [7]. Interestingly, microbial metabolites play the role of messengers during host–microbiota interactions [8]. For example, microbial aromatic amino acid metabolites act as signaling molecules during biological processes, including immune homeostasis. Thus, these microbial aromatic amino acid metabolites are promising therapeutic targets in animal autoimmune disease models, including models of inflammatory bowel disease and multiple sclerosis [9].

Usually, germ-free mice are used to explore the role of the microbiota in host physiology. However, this model is not easy to access for many researchers due to a requirement for specialized facilities, high costs, and complex and labor-intensive techniques. Therefore, a cocktail of broad-spectrum antibiotics has been developed to regenerate microbiota-depleted mice easily [10]. The combination of ampicillin, vancomycin, neomycin, and metronidazole is one of the most common cocktails utilized to achieve this [11,12,13]. Previous studies have shown the effects of each of these antibiotics on physiological activities such as metabolism and immunity, as well as microbiota composition in normal or diseased mice models [14,15,16]. However, studies on the association between specific bacterial species and physiologic markers altered by antibiotic administration are still scarce.

Therefore, the aim of this study was to investigate the effect of each of these antibiotics on host intestinal metabolism and on expression of immune-related genes, as well as their effects on the gut microbiota. Mice received one of four antibiotics (ampicillin (AMP), vancomycin (VAN), neomycin (NEO), and metronidazole (MET)) or a cocktail of all four (AVNM) via drinking water. First, we sought to study the effects of each antibiotic on (i) the composition of fecal microbiota, (ii) intestinal microbial metabolite profiles, and (iii) host immune-related gene expression in the gut. Next, we examined the association between antibiotic treatment-induced gut microbiota changes and the changes in intestinal metabolic profiles and immunological gene expression.

## 2. Results

### 2.1. Antibiotic-Induced Changes in the Diversity and Structure of the Fecal Bacteria Community

**Figure 1:**
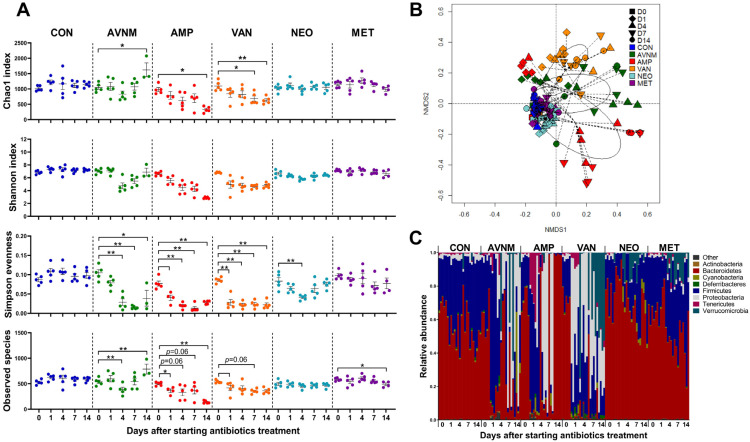
Effects of antibiotic treatment on fecal microbiota in mice. (**A**) Changes over time of alpha diversity in each antibiotic group. (**B**) NMDS plot of the fecal microbiota structure with Bray–Curtis distance. (**C**) Microbial composition comparison at phylum level. Data are expressed as the mean ± SEM. Asterisks indicate a statistically significant difference (* *p* < 0.05, ** *p* < 0.01; Mann–Whitney U test, CON, control; AVNM, mixture of ampicillin, vancomycin, neomycin and metronidazole; AMP, ampicillin; VAN, vancomycin; MET, metronidazole).

First, we wondered whether different microbiota communities are formed in the presence of different antibiotics. Each group of mice was treated with antibiotics for 14 days. There was no significant difference in the average water intake per individual mouse per day in any group (Supplementary Figure S1). We analyzed fecal samples from each antibiotic-treated mouse immediately before treatment (Day 0), and then again on Days 1, 4, 7, and 14 after treatment. The alpha diversity of the fecal microbiota at each time point was assessed using the Chao1, Shannon, Simpson evenness, and Observed Species indices. We observed distinct alterations in alpha diversity in the AMP- and VAN-treated groups compared with other groups. AMP and VAN induced significant decreases at all the measurement days only in the Shannon and Simpson evenness indices. Alpha diversity changed little in the neomycin NEO- and MET-treated groups. AVNM treatment induced a steady decrease only in the Simpson evenness index, while other parameters tended to recover from Day 7 (Figure 1A).

Changes in the beta diversity of the fecal microbiota were visualized in an NMDS plot based on the Bray–Curtis dissimilarity distance. The fecal microbiota in the AMP, VAN, and AVNM groups changed markedly from Day 1 of antibiotic treatment (Figure 1B and Supplementary Figure S2).

To observe compositional changes, the fecal microbiota of each group were compared at the phylum level. Phylum composition changed from Day 1 of treatment. Compositional changes were most conspicuously observed in AMP, VAN, and AVNM at the phylum level on Day 14 (Figure 1C).

### 2.2. Major Bacterial Alterations under Antibiotic Treatment

**Figure 2:**
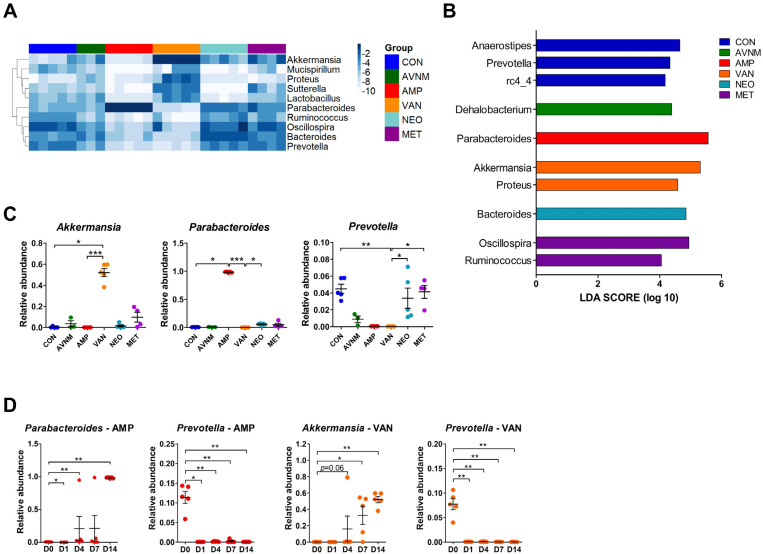
Dominant taxa comparison of each antibiotic group on Day 14 after the start of antibiotic treatment. (**A**) Heatmap of -log of relative abundance of top 10 abundant genera. (**B**) Histograms of the linear discriminant analysis (LDA) scores for abundant genus in each antibiotic-treated group. (**C**) Relative abundance of major discriminative taxa in AMP and VAN groups. (**D**) Relative abundance of *Parabacteroides*, *Prevotella*, and *Akkermansia* by date from antibiotic treatment. Data are expressed as the mean ± SEM. Asterisks indicate a statistically significant difference (* *p* < 0.05; ** *p* < 0.01; *** *p* < 0.001 Kruskal–Wallis one-way analysis of variance with the Dunn’s post hoc test. The LDA score cut-off was set to 2.0; CON, control; AVNM, mixture of ampicillin, vancomycin, neomycin and metronidazole; AMP, ampicillin; VAN, vancomycin; MET, metronidazole).

To determine the dominant taxa in each treatment group, we analyzed the fecal microbiota at the genus level. The heatmap shows the top 10 most abundant genera on Day 14 after the start of antibiotic treatment. Similar to the microbiota community (see Figure 1), AMP and VAN had the greatest effect on genus composition (Figure 2A). In particular, we observed a marked increase in the abundance each of *Parabacteroides* in the AMP group and of *Akkermansia* in the VAN group, whereas a distinctly decreased abundance of *Prevotella* was observed in both groups (Figure 2A). LDA effect size (LEfSe) analysis revealed that *Prevotella* and rc4-4 in the CON group, *Parabacteroides* in the AMP group, *Akkermansia* and *Proteus* in the VAN group, *Bacteroides* in the NEO group, *Oscillospira* and *Ruminococcus* in the MET group, and *Dehalobacterium* in the AVNM group were the significantly dominant genera (Figure 2B). In particular, *Parabacteroides* in the AMP group and *Akkermansia* in the VAN group were the most dominant (LDA score > 5.0).

Additionally, these results were replicated in the Kruskal–Wallis test. In the AMP-treated group, *Parabacteroides* increased, but *Oscillospira* and *Ruminococcus* decreased. VAN treatment boosted *Akkermansia* and *Proteus*, and reduced *Prevotella* and *Ruminococcus* (Figure 2C and Supplementary Figure S3). The significant increase in the abundance of *Parabacteroides* and *Akkermansia* in the AMP and VAN groups, respectively, was observed from Day 4. However, the abundance of *Prevotella* fell immediately (Day 1) (Figure 2D and Supplementary Figure S4).

### 2.3. Antibiotic-Induced Changes in Gut Metabolites

**Figure 3:**
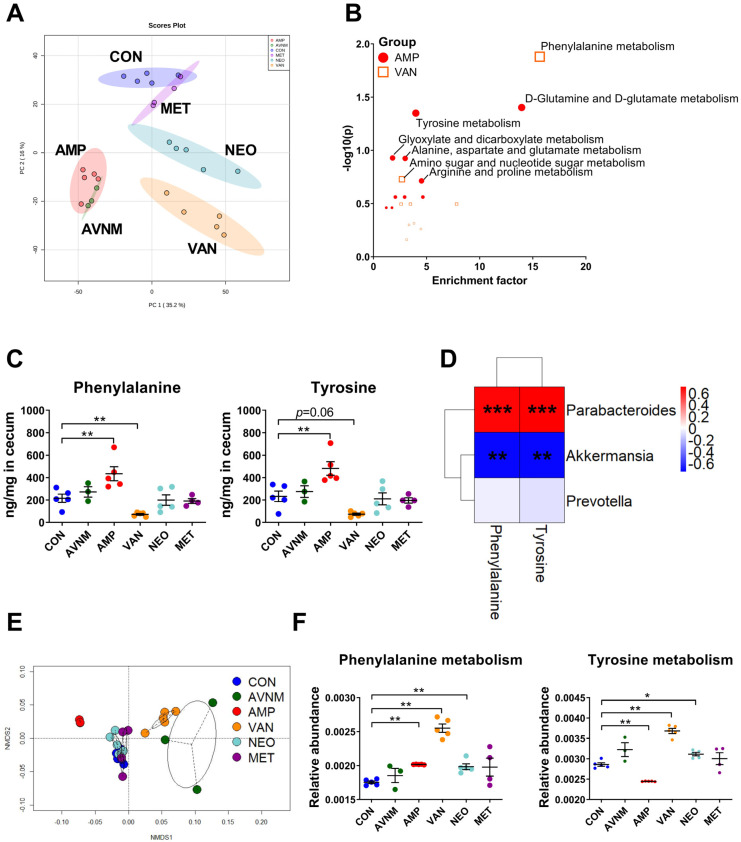
Effects of antibiotic treatment on gut metabolites and correlation with the gut microbiota. (**A**) The principal component analysis (PCA) score plot of untargeted mass spectrometry data in cecal contents of each group. (**B**) Enriched pathway in cecal metabolite of AMP and VAN group. (**C**) The quantity of phenylalanine and tyrosine in cecal content. (**D**) The Spearman’s correlation analysis between relative abundance of major discriminative taxa and the quantity of amino acids in significantly enriched pathways in AMP and VAN group. (**E**) NMDS plot of the predicted function of fecal microbiota with Bray–Curtis distance. (**F**) The relative abundance of predicted function of phenylalanine and tyrosine metabolism in fecal microbiota. Data are expressed as the mean ± SEM. Asterisks indicate a statistically significant difference (* *p* < 0.05, ** *p* < 0.01, *** *p* < 0.001; Mann–Whitney U test and Spearman’s correlation, CON, control; AVNM, mixture of ampicillin, vancomycin, neomycin and metronidazole; AMP, ampicillin; VAN, vancomycin; MET, metronidazole).

Given that alterations in the gut microbiome composition and microbial metabolic changes influence the host’s metabolism, we performed non-targeted metabolomics analysis. Although metabolite composition in all antibiotic-treated groups was distinguishable from that in the control group, the AMP, VAN, and AVNM groups had more diverse compositions than the NEO and MET groups (Figure 3A and Supplementary Figure S5). To confirm that these metabolomic compositional alterations are related to specific metabolic pathways, enriched metabolic pathways were assumed in each AMP and VAN group through functional analysis based on the KEGG pathway at the Metaboanalyst website (Figure 3B). In the AMP group, D-glutamine and D-glutamate metabolism and tyrosine metabolism pathways were enriched significantly, whereas the phenylalanine metabolism pathway was enriched in the VAN group. In parallel, the amount of tyrosine and phenylalanine was significantly different between the AMP and VAN groups and the CON group; AMP increased the amount of both amino acids in the cecum, while VAN significantly decreased the amount of phenylalanine and had a tendency to decrease the amount of tyrosine (Figure 3C).

The amount of most amino acids increased in the AMP group but decreased in the VAN group. In the AMP group, the amount of threonine, serine, tyrosine, lysine, methionine, phenylalanine, isoleucine, leucine, and valine increased. By contrast, the amount of arginine, tyrosine, lysine, methionine, phenylalanine, isoleucine, and leucine decreased (Supplementary Figure S6).

Considering that the gut microbiota is involved in host amino acid metabolism [17], we performed Spearman’s correlation analysis to see if there was a correlation between the abundance of particular genera and the amount of amino acids (Figure 3D). The amount of tyrosine and phenylalanine correlated positively with the abundance of *Parabacteroides*, the major taxa in the AMP group. Moreover, there was a negative correlation between the amount of these amino acids and the abundance of *Akkermansia*, which was dominant in the VAN group. However, there was no significant correlation between the amount of these amino acids and the abundance of *Prevotella*, which was decreased in the AMP and VAN-treated groups (Figure 2C and Figure 3D, and Supplementary Figure S7).

To clarify that these associations were induced by changes in the gut microbiota after antibiotic treatment, we performed PICRUSt analysis, which shows potential changes in the functions of the gut microbiota (Figure 3E,F). The NMDS plot revealed that differences in the potential function of the gut microbiota occurred only in the AMP, VAN, and AVNM groups (Figure 3E). In parallel with the enriched pathway of metabolomics data observed in Figure 3B,C, phenylalanine and tyrosine metabolism by gut microbiota was altered significantly in the AMP and VAN groups (Figure 3F).

### 2.4. Changes in Expression of Immune-Related Genes in the Gut in Response to Antibiotic Treatment

**Figure 4:**
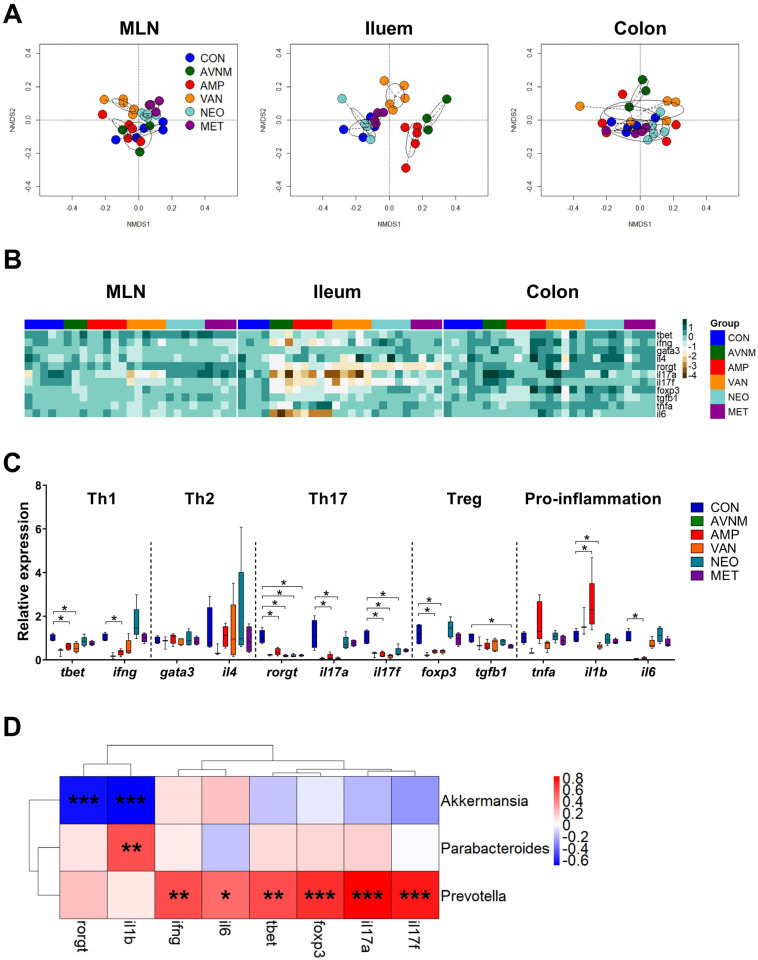
Effects of antibiotic treatment on immune-related gene expression in the gut and correlation with the gut microbiota. (**A**) NMDS plots of the fold expression of gene expression in MLN, ileum and colon with Bray–Curtis distance. (**B**) Heatmap of -log of the fold expression in MLN, ileum and colon. (**C**) The relative expression of genes related to Th1, Th2, Th17, Treg, and pro-inflammation in ileum. (**D**) The Spearman’s correlation analysis between relative abundance of major discriminative taxa and the relative expression of genes significantly changed to CON in AMP and VAN groups. Data are expressed as the mean ± SEM. Asterisks indicate a statistically significant difference (* *p* < 0.05, ** *p* < 0.01, *** *p* < 0.001; Mann–Whitney U test and Spearman’s correlation, CON, control; AVNM, mixture of ampicillin, vancomycin, neomycin and metronidazole; AMP, ampicillin; VAN, vancomycin; MET, metronidazole).

The gut microbiota play a major role in the development of gut immunity; thus, we investigated whether antibiotic administration affects the expression of genes related to immunity in the intestine. To analyze the intestine overall, we extracted RNA from MLN, ileum, and colon, and categorized target genes as helper T cell (Th) 1-, Th2-, Th17-, Treg-, and pro-inflammatory-related. The NMDS and heatmap plots revealed that changes in gene expression were more pronounced in the AMP, VAN, and AVNM groups than in the CON, NEO, and MET groups, which was similar to the observations regarding the microbiome (Figure 1B and Supplementary Figure S2). However, the change in immunological gene expression was more pronounced in the ileum than in the MLN and colon (Figure 4A,B, and Supplementary Figure S8). With the exception of Th2-related genes, such as *gata3* and *il4*, there were significant reductions in the expression of most genes in the AMP and VAN groups; only the expression of *il1b*, a pro-inflammation-related gene, was increased in the AMP group (Figure 4B,C). In particular, the expression of Th17-related genes, including *rorgt*, *il17a*, and *il17f* was most affected by antibiotic treatment; the expression of those genes was decreased in all antibiotic treated groups (Figure 4B,C).

To determine whether these changes in gene expression are associated with the gut microbiota, we performed Spearman’s correlation analysis to assess the correlation between genes that showed significant changes in expression in the AMP and VAN groups and the abundance of *Parabacteroides*, *Akkermansia*, and *Prevotella* (Figure 4D). Interestingly, the amounts of amino acids were correlated with the abundance of *Akkermansia*, which was increased in the VAN group, and *Parabacteroides*, which was increased in the AMP group (Figure 3D) whereas ileal immunological gene expression was mainly associated with the abundance of *Prevotella*, the declined genus in both AMP and VAN groups (Figure 4D).

## 3. Discussion

Gut microbiota perform diverse functions, including the regulation of host metabolism and immunity [18]. As such, the diversity of the gut microbiota is important for health [19]. However, intestinal microbial dysbiosis can be caused by many environmental factors, including antibiotic use. Antibiotics are used to treat infectious diseases. As each antibiotic has a particular mode of action, their prescription is targeted at particular classes of microbe. Therefore, it is essential to study changes in host physiology in response to individual antibiotics, because each can have a different effect on host metabolism and immune responses. In this study, we examined changes in the microbiome, gut metabolites, and expression of genes related to immune responses in mice treated with AMP, VAN, NEO, MET, or a cocktail of all four antibiotics.

First, the most distinct alterations in alpha and beta diversity were observed in the groups treated with AVNM, AMP or VAN; these changes were observed immediately after the start of antibiotic treatment. The abundance of *Parabacteroides* and *Akkermansia* increased in the AMP and VAN groups, respectively, as observed in other studies [20,21], which could be due to bacterial resistance to these antibiotics [20]. *Parabacteroides distasonis* is resistant to AMP, a β-lactam antibiotic, by producing β-lactamase [22]. *Akkermansia muciniphila* is initially resistant to VAN because VAN with a molecular weight more than 1400 Da cannot pass through the outer membrane of gram-negative bacteria [23,24]. However, in silico gene prediction analysis also revealed that a strain of *Akkermansia muciniphila* expresses vancomycin-resistance genes, including the glycopeptide vanX [25,26]. Although to a lesser extent, changes in the microbial composition were also observed in the neomycin-treated group. In particular, an abundance of *Bacteroides* was increased in the NEO group, which was also observed in a previous study on the effect of neomycin on the gut microbiota [27]. This could be due to the incapability of *Bacteroides* in carrying out oxygen- or nitrate-dependent electron transport, thereby failing to transport aminoglycosides, to which neomycin belongs [28]. Additionally, in the group treated with AVNM, distinct compositional changes in the gut microbiome were also observed as in the AMP and VAN groups. Interestingly, the changes in the alpha diversity showed different results depending on the index. This tendency is also observed in a previous study [16], which may be due to the difference in the calculation method for each alpha-diversity index [29]. For example, Observed species is an index indicating richness and Simpson evenness is an index indicating evenness, while the Shannon index is an index calculated considering both richness and evenness. Therefore, the results of this study indicate that index types of alpha diversity should be determined according to the purpose of each study. On the other hand, MET hardly showed an alteration in the gut microbial community, which has also been reported in previous papers [30,31]. This is likely due to the fact that MET is absorbed mainly in the small intestine; thus it does not reach the ileum, cecum, or colon [32].

The gut microbiota composition, as well as microbial metabolism, can contribute to host metabolism [5]. We discovered that pathways related to amino acid metabolism were enriched in the intestine of antibiotic-treated mice; also, the amount of tyrosine and phenylalanine, aromatic amino acids, was positively correlated with the abundance of *Akkermansia* and *Parabacteroides* in the VAN and AMP groups. Interestingly, a ketogenic diet has a marked effect on amino acid metabolism, increasing the abundance of *Akkermansia* and *Parabacteroides* [33]. Thus, these two taxa may be closely related to aromatic amino acid metabolism in response to environmental changes. Interestingly, recent studies have reported that the metabolism of aromatic amino acids by gut microbiota acts as a signal for communication between the host and the microbiota [9]. Production of tryptophan, phenylalanine, and tyrosine (all aromatic amino acids) is regulated by the gut microbiota, and changes in the circulating concentration of these amino acids affect gut permeability and systemic immunity [9]. Additionally, phenylalanine is converted to tyrosine by phenylalanine hydroxylase (PAH) in a healthy human subject. Tyrosine is further used as a precursor to neurotransmitters such as epinephrine, norepinephrine, and dopamine [34,35]. Thus, the phenylalanine metabolism plays an important role in mental health. On the other hand, phenylketonuria, an inborn disease or kidney disorder, impairs the function of the phenylalanine metabolism [36]. Therefore, these results suggest that the types of antibiotics for patients with impaired amino acid metabolism should be considered.

Our study showed that each type of antibiotic affected immune responses in the MLN, ileum, and colon. The majority of changes were observed in the ileum, an immunologically active compartment in the gut [37]. However, colonic immunity should not be overlooked, as it also plays an important role in the host, different to that of the immune system of the small intestine. The colon is the reservoir for huge numbers of commensal microorganisms [38]. Therefore, the immune system of the colon recognizes these microbiota and concentrates on maintaining an appropriate distance from the host. This involves the production of a thick mucus layer, the generation of IgA antibodies and the presence of large numbers of regulatory T cells [39]. In the present study, only RNA expression of cytokine was confirmed without investigating the overall immune responses in the colon. Further studies are needed through wider measurement experiments for the precise effects of antibiotics on colonic immunity.

Among helper T cells, antibiotic administration had the greatest effect on intestinal Th17-related immune responses. This phenomenon was also observed in other studies [3]. However, dampened Th17-related immune responses in antibiotic-treated mice are also observed in other organs such as the lung [40,41] and spleen [41]. These previous studies show a correlation between Th17-mediated immunity and segmented filamentous bacteria. However, since there are many other abundant immune-related bacteria in the gut, it is necessary to examine the role of these bacteria in the reduction of Th17 responses upon treatment with antibiotics. In this context, our study showed an additional major association between *Prevotella* and immune responses. Indeed, previous studies show that *Prevotella* induces Th17 responses [42]. In addition to Th17-mediated responses, including the expression of *il17a* and *il17f*, we found that antibiotic-induced changes in immunological gene expression, such as those of *ifng*, *il6*, *tbet*, and *foxp3*, were closely related to the abundance of *Prevotella*, raising the possibility that *Prevotella* might be one of most influential genera involved in the control of intestinal immunity. Th17-related immune responses have been involved in the pathogenesis of autoimmune diseases such as rheumatoid arthritis (RA) and inflammatory bowel disease (IBD) [43]. On the other hand, Th17 cells are also known to regulate protective immunity against various pathogens, including *Mycobacteria tuberculosis* or *Klebsiella pneumonia* [44]. Given that administration of AMP and VAN reduced the expression of Th17-related genes in the present study, administration of these antibiotics may be a double-edged sword depending on the immune pathogenesis of various diseases. Therefore, not only the mode of action but also these immune effects of antibiotics should be considered when administering antibiotics to patients with these immune diseases.

However, there are some limitations in this study. First of all, there is an issue that some antibiotics such as metronidazole may induce avoidance to drinking water containing the antibiotics, which, in turn, induces the death of mice [45]. Thus, the information on basic health conditions such as body weight needs to be provided. Moreover, this study was conducted using a small size of animal numbers per group. Further studies using increased numbers of mice are required to improve the reliability of the findings. In addition, our study analyzed intestinal microbiomes using 16S rRNA gene amplicon sequencing, which gives us an information based on the relative abundance. Considering the fact that the antibiotics can inhibit the growth of bacteria, the total number of bacteria needed to be measured. Moreover, metagenomic shotgun sequencing would be required for better species-level resolution. It is also necessary to confirm the actual protein levels of immunological genes considering the unreliability of mRNA. Finally, this study focused on biological changes observed in intestines. Further studies about systemic changes on immune profiles would be valuable, because gut microbiota could affect other organs.

In conclusion, we revealed that antibiotic administration affected both the gut microbiota and host physiology, including amino acid metabolism and immunity. The magnitude of these effects depended on different types of antibiotics. Moreover, there was an association between altered gut microbiota and host physiology. Thus, our results provide a basis for guidelines for antibiotics treatment, especially for patients with metabolic and/or immune diseases. In addition, bacteria associated with specific host physiology could be a good target for such diseases.

## 4. Materials and Methods

### 4.1. Mice and Antibiotic Treatment

Female BALB/c mice (6 weeks old) were purchased from Orient Bio, Inc. (Seongnam, Republic of Korea) and housed in a conventional laboratory animal facility at the Research Institute of Pharmaceutical Sciences at Seoul National University, according to the experimental animal use guidelines of Seoul National University. All experimental protocols were approved by the Seoul National University Institutional Animal Care and Use Committee (IACUC: SNU-180104-2-3). The experimental groups were divided into six groups, namely a control group (no antibiotic treatment; CON, and N = 5) and five antibiotic treatment groups (AMP, VAN, NEO, MET, and AVNM). The characteristics of each antibiotic used in this study are summarized in Table 1. For antibiotic treatment, mice received autoclaved drinking water supplemented with 1 g/L AMP, 0.5 g/L VAN, 1 g/L NEO, 1 g/L MET, or an AVNM cocktail consisting of 1 g/L AMP, 0.5 g/L VAN, 1 g/L NEO, and 1 g/L MET for 2 weeks (N = 5 per group; N = 3 for AVNM group and N = 4 for MET group at Day 14 due to death). These concentrations were based on the literature [46]. All antibiotics were purchased from Sigma-Aldrich (St. Louis, MO, USA). Water consumption was monitored every 3–4 days during the treatment period. Fecal samples were collected immediately before antibiotic treatment, and on Days 1, 4, 7, and 14 after treatment. The provided diet was normal laboratory chow purchased from Purina (Seongnam, Korea; contents: crude protein = 20.0%, crude fat = 4.5%, crude fiber = 6.0%, ash = 7.0%, and added minerals = 2.5%). On Day 14 after the start of antibiotic administration, mice were euthanized, and MLN, ileum, colon, and cecum were harvested. Feces and tissue samples were stored at −80 °C for further analyses.

### 4.2. Microbiome Analysis Using 16S rRNA Gene Amplicon Sequencing

DNA was extracted from fecal sample suspensions using the QIAamp DNA Fecal Mini Kit (Qiagen, Hilden, Germany) and stored at −20 °C until use. The V4 region of the 16S rRNA gene was amplified from fecal DNA using Illumina-adapted universal primers 515F/806R (515 F: forward primer, 5′-AATGATACGGCGACCACCGAGATCTACACTATGGTAATTGTGTGCCAGCMGCCGCGGTAA-3′; 806 R: reverse primer containing a unique 12-base golay barcode for tagging each polymerase chain reaction [PCR] product, 5′-CAAGCAGAAGACGGCATACGAGATNNNNNNNNNNNNAGTCAGTCAGCCGGACTACHVGGGTWTCTAAT-3′). The amplicons were pooled and sequenced using the MiSeq platform (Illumina, San Diego, CA, USA). PCR amplicons were purified using the QIAquick PCR Purification Kit (Qiagen), and quantified using the KAPA Library Quantification Kit (KAPA Biosystems, Woburn, MA, USA) and an ABI 7300 (Applied Biosystems, Carlsbad, CA, USA) machine.

Sequence data were analyzed using the QIIME software package (version 1.8.0) [47]. Closed-reference OTU picking was performed at 97% sequence similarity, based on the gg_13_5 Greengenes database. Representative sequence sets were chosen using UCLUST, and processed sequences were aligned using PyNAST. Taxonomy was assigned using the ribosomal database project classifier, with the minimum confidence score for taxonomy assignment to sequences set to 0.8. Chimeric sequences were excluded from downstream analyses prior to the generation of OTU tables using the ChimeraSlayer algorithm. OTUs were rarefied to 4000 sequences. Rarefied OTUs were collapsed at the genus level.

Alpha diversity within the microbial communities of each treatment group was measured using the Chao1, Shannon, Simpson evenness, and Observed species indices. Analysis of beta diversity was based on Bray–Curtis distance. Heatmaps and nonmetric multidimensional scaling (NMDS) plots were generated using R packages pheatmap and vegan, respectively. The linear discriminant analysis (LDA) effect size (LEfSe) algorithm was run using the Galaxy application tool (http://huttenhower.sph.harvard.edu/galaxy/ accessed on 15 July 2017), with a linear discriminant analysis cut-off score of 2.0 and a *p*-value of <0.05.

### 4.3. LC-QTOF-MS/MS Analysis

Cecal contents were weighed, and 80% methanol was added at a 1:1 ratio to extract metabolites. This was followed by sonication for 3 min until the samples were fully homogenized. After centrifugation at 16,000× *g* for 1 min, the supernatant was passed through a 0.22 μm filter. The filtered supernatant was then evaporated in a Speedback (Eppendorf, Germany). Finally, metabolites within the cecal contents were obtained in powder form and stored at −80 °C until analysis.

Amino acids in the cecal samples were measured as previously described [48]. Amino acid derivation was conducted using AccQ•Tag reagents (Waters Corporation, Milford, MA, USA). Amino acid separation was performed using an ACQUITY UPLC^®^ HSS T3 column (100 mm × 2.1 mm, 1.7 m) and an Acquity UPLCTM system (Waters Corporation). The analysis conditions for chromatography were as follows: mobile phase A = water with 0.1% formic acid; mobile phase B = acetonitrile with 0.1% formic acid; injection volume = 4 μL. The time gradient of the mobile phase was maintained at 4% B at 0.5 min, increasing to 10% B at 2.5 min, 28% B at 5 min, and 95% B at 5.1 min, and reverting to 4% B at 6.2 min for a 1.3 min re-equilibration. The flow rate was 0.6 mL/min. Qualitative amino acid analysis was conducted using a Waters Synapt G2-Si Q-TOF mass spectrometer (Waters Corporation) equipped with an electrospray (ESI) probe in positive ionization mode and MRM mode. For the Synapt G2-Si QTOF, the following mass spectrometer parameters were applied: capillary, 25 kV; source temperature, 100 °C; sampling cone, 40; source offset, 80; desolvation temperature, 250 °C; cone gas flow, 50 L/h; desolvation gas flow, 600 L/h; nebulizer gas flow, 6.5 Bar. Each measured amino acid was quantified using the QuanLynx of MassLynx program [49].

The untargeted metabolites analysis was based on a previous study [50]. Measurement of total untargeted metabolites was performed using an Acquity UPLC BEH C18 column under UPLC conditions and in positive ionization mode and MSe scan mode. The mass range was set from 50 to 1200 Da, and the scan time was set to 0.2 s. The following mass spectrometer parameters were used: capillary, 2 kV; source temperature, 120 °C; sampling cone, 40; source offset, 80; desolvation temperature, 400 °C; cone gas flow, 50 L/h; desolvation gas flow, 600 L/h; nebulizer gas flow, 6.5 bar. The identity of the amino acids was confirmed by alignment to the AA-S-18 analytical standards mixture (Sigma-Aldrich). All systems were controlled by Mass-LynxTM software 4.1 (Waters Corporation) [49].

Intergroup metabolism analysis was carried out by transferring MassLynxTM software to Progenesis QI software (Waters Corporation). A retention time window of 0.20 min and a mass tolerance of 1.0 ppm were set to align the compounds. Then, ANOVA P-values and max fold changes were used to filter compounds. The final data were exported to Metaboanalyst for metabolome analysis.

### 4.4. Gene Expression Analysis

MLN, ileum, and colon samples were homogenized with a stainless-steel bead (diameter, 5 mm; Qiagen) for 5 min at 30 Hz. Total RNA was isolated using an Easy-spin Total RNA extraction kit (Intron, Seoul, Republic of Korea). Next, cDNA synthesis was performed using the High-Capacity RNA-to-cDNA Kit (Applied Biosystems). Expression of genes (*tbet, ifng, gata3, il4, rorgt, il17a, il17f, foxp3, tgfb1, tnfa, il1b,* and *il6*) was estimated using the Rotor-Gene SYBR Green PCR Kit (Qiagen) and the Rotor-Gene Q cycler (Qiagen). The primers used for qPCR are listed in Supplementary Table S1. The 2-ΔΔCt method was used for relative quantification of gene expression. Finally, mRNA gene expression was normalized against *gapdh* expression.

### 4.5. Statistical Analysis

Statistical analysis of non-targeted metabolic data in MetaboAnalyst for principal component analysis (PCA) was performed with the following conditions: normalization by median, Log transformation, and auto scaling. The other statistical analyses were performed using Prism 5 (GraphPad Software, San Diego, CA, USA). When comparing two groups, statistical significance was measured using the Mann–Whitney test. Statistical comparisons of relative abundance of genera and average water intake in each group were analyzed using Kruskal–Wallis one-way ANOVA, followed by Dunn’s post hoc test. In all graphs, data are presented as the mean ± standard error of the mean (SEM). * *p* < 0.05, ** *p* < 0.01, and *** *p* < 0.001 denote statistical significance.

## Figures and Tables

**Table 1:** Characteristics of antibiotics used in this study.

Antibiotics	Abbreviation	Class	Spectrum of Activity
Ampicillin	AMP	β-lactams	Gram-positives and -negatives
Vancomycin	VAN	Glycopeptide	Gram-positives
Neomycin	NEO	Aminoglycoside	Gram-negatives
Metronidazole	MET	Anaerobic DNA inhibitor	Anaerobes

## Data Availability

The V4 16S rDNA sequence datasets obtained from this study have been deposited in the European Nucleotide Archive databases with the accession codes ERP142675 (https://www.ebi.ac.uk/ena/browser/view/PRJEB57688).

## Supplementary Materials

The following supporting information can be downloaded at: https://www.mdpi.com/article/10.3390/antibiotics11121762/s1, Figure S1: Average water intake per individual mouse per day. Figure S2: Changes of gut microbial community according to the date from antibiotic treatment. Figure S3: Relative abundance of top 10 abundant genus except for genus shown in Figure 2C on day14 after treatment. Figure S4: Changes of relative abundance of top 10 abundant genus according to the date from antibiotic treatment. Figure S5: Heatmap of non-targeted metabolites in cecal content in each antibiotic group on day14. Figure S6: The quantification of amino acids in cecum samples in each antibiotic group on day14 after treatment. Figure S7: The individual spearman’s correlation analysis between relative abundance of major discriminative taxa and the quantity of amino acids in significantly enriched pathways in AMP and VAN group. Figure S8: The relative gene expression in MLN and Colon in each antibiotic group on day14. Table S1: LC-QTOF-MS data in cecum in each antibiotic group on day14.
