# Supplementary material for: Distinct Changes in Microbiota-Mediated Intestinal Metabolites and Immune Responses Induced by Different Antibiotics
Source: Antibiotics (Basel). 2022 Dec 6;11(12):1762. doi: 10.3390/antibiotics11121762 (PMC9774394; doi:10.3390/antibiotics11121762)

Supplementary Figure S1. Average water intake per individual mouse per day. Average water intake was measured four times every 4 days. Statistical significance was analyzed by Kruskal-Wallis test. CON, control; AVNM, mixture of ampicillin, vancomycin, neomycin and metronidazole; AMP, ampicillin; VAN, vancomycin; MET, metronidazole.

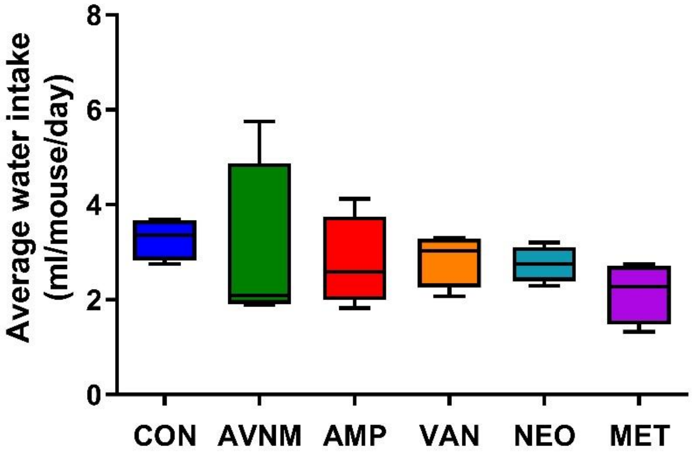

Supplementary Figure S2. Changes of gut microbial community according to the date from antibiotic treatment. NMDS plot of the fecal microbiota structure with Bray-Curtis distance on right before treatment (day 0), as well as at day1, day4, day7, and day14 after antibiotic treatment. CON, control; AVNM, mixture of ampicillin, vancomycin, neomycin and metronidazole; AMP, ampicillin; VAN, vancomycin; MET, metronidazole.

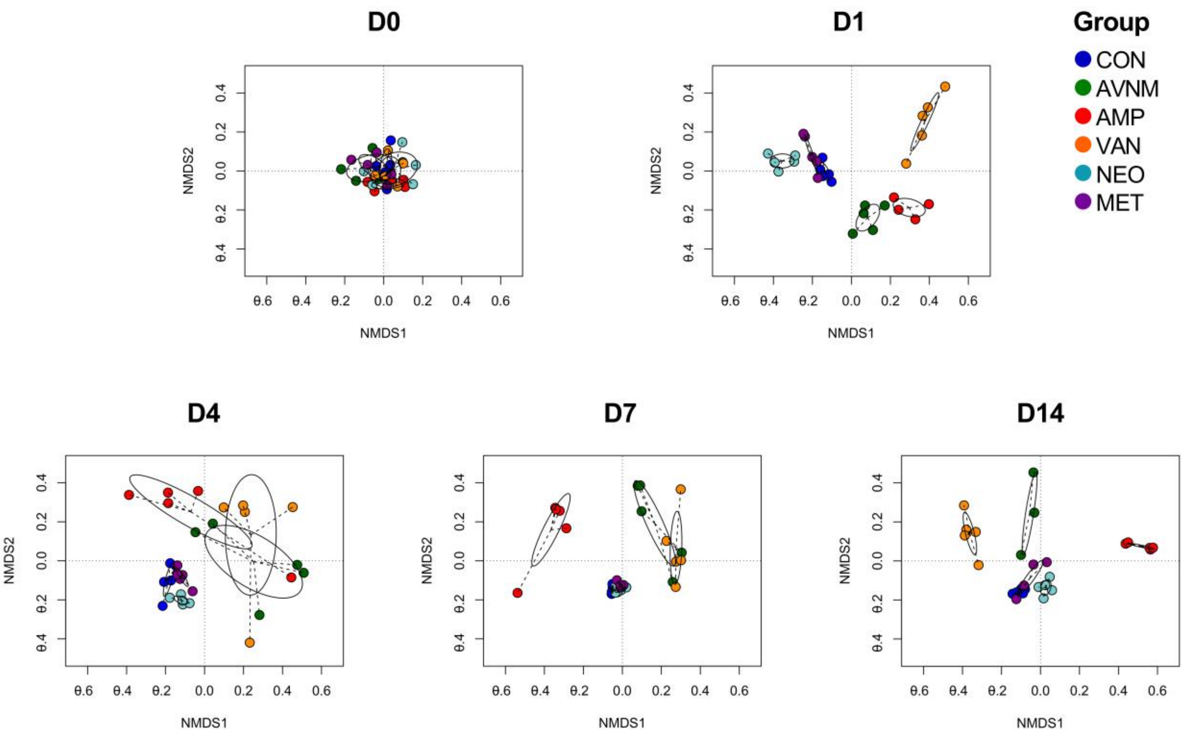

Supplementary Figure S3. Relative abundance of top 10 abundant genus except for genus shown in Fig. 2C on day14 after treatment. Relative abundance of Proteus, Sutterella, Lactobacillus, Mucispirillum, Ruminococcus, Bacteroides, and Oscillospira. (*P < 0.05; **P < 0.01, ***P < 0.001; Kruskal-Wallis one-way analysis of variance with the Dunn's post-hoc test. The LDA score cut-off was set to 2.0, CON, control; AVNM, mixture of ampicillin, vancomycin, neomycin and metronidazole; AMP, ampicillin; VAN, vancomycin; MET, metronidazole).

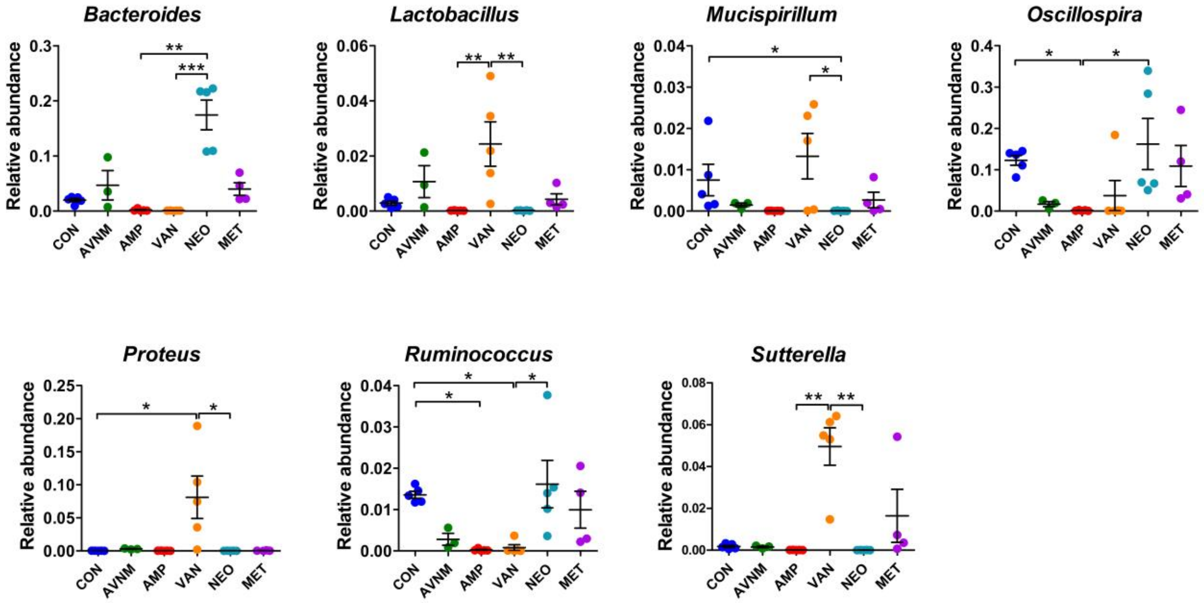

Supplementary Figure S4. Changes of relative abundance of top 10 abundant genus according to the date from antibiotic treatment. Heatmap of -log of relative abundance of top 10 abundant genus on day0, day1, day4, day7, and day14 after treatment in each antibiotic-treated group. Red boxes show changes of relative abundance over time of major discriminative taxa in AMP and VAN group shown in Fig. 2C. CON, control; AVNM, mixture of ampicillin, vancomycin, neomycin and metronidazole; AMP, ampicillin; VAN, vancomycin; MET, metronidazole.

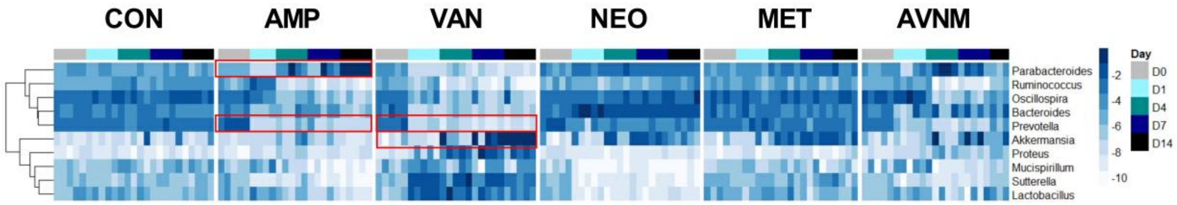

Supplementary Figure S5. Heatmap of non-targeted metabolites in cecal content in each antibiotic group on day14. Non-target metabolites were clustered. CON, control; AVNM, mixture of ampicillin, vancomycin, neomycin and metronidazole; AMP, ampicillin; VAN, vancomycin; MET, metronidazole.

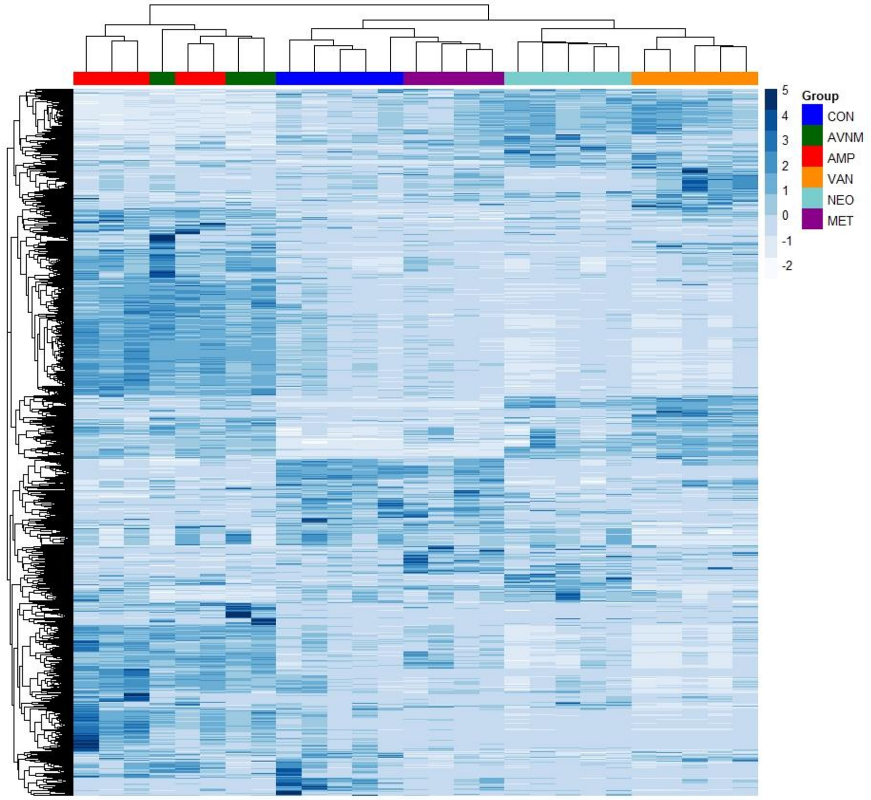

Supplementary Figure S6. The quantification of amino acids in cecum samples in each antibiotic group on day14 after treatment. The quantity of amino acids in cecal content except for genus shown in Fig. 3C. (*P < 0.05, **P < 0.01; Mann-Whitney U test, CON, control; AVNM, mixture of ampicillin, vancomycin, neomycin and metronidazole; AMP, ampicillin; VAN, vancomycin; MET, metronidazole).

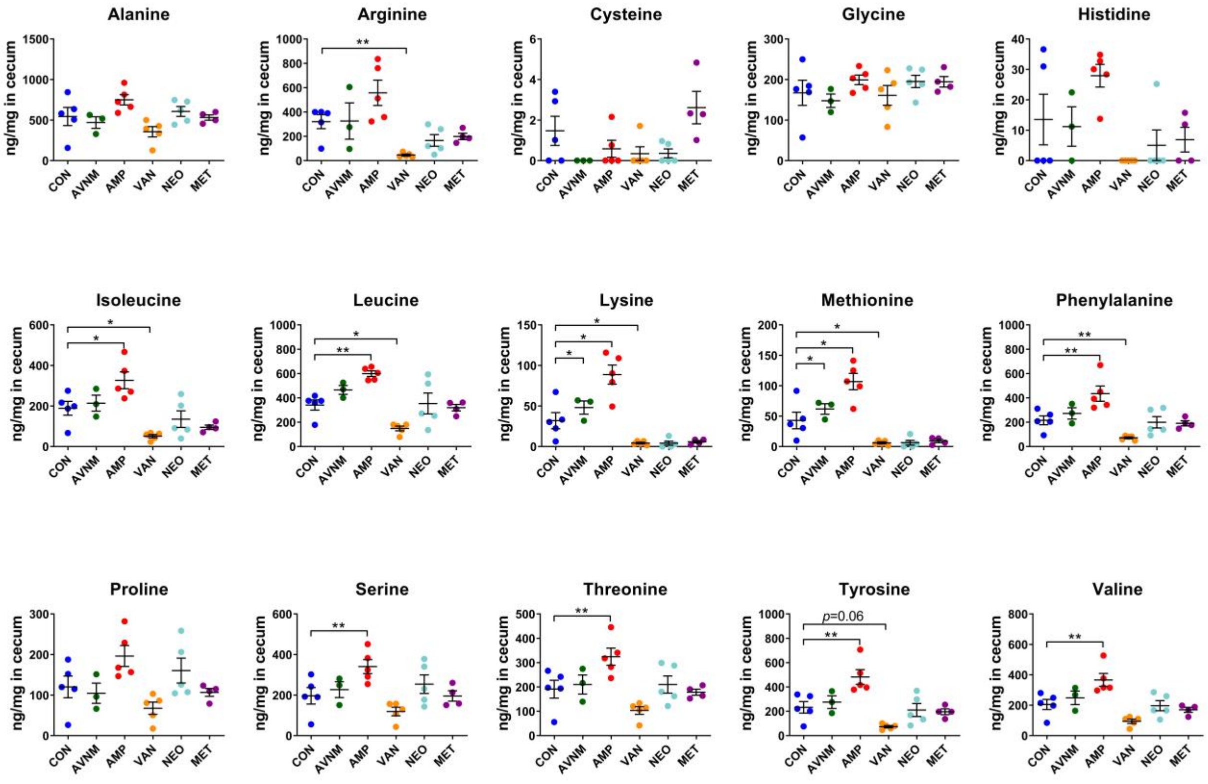

Supplementary Figure S7. The individual spearman's correlation analysis between relative abundance of major discriminative taxa and the quantity of amino acids in significantly enriched pathways in AMP and VAN group. The spearman's correlation between fecal relative abundance of *Parabacteroides*, *Akkermansia*, or *Prevotella* and cecal concentration of Tyrosine or Phenylalanine.

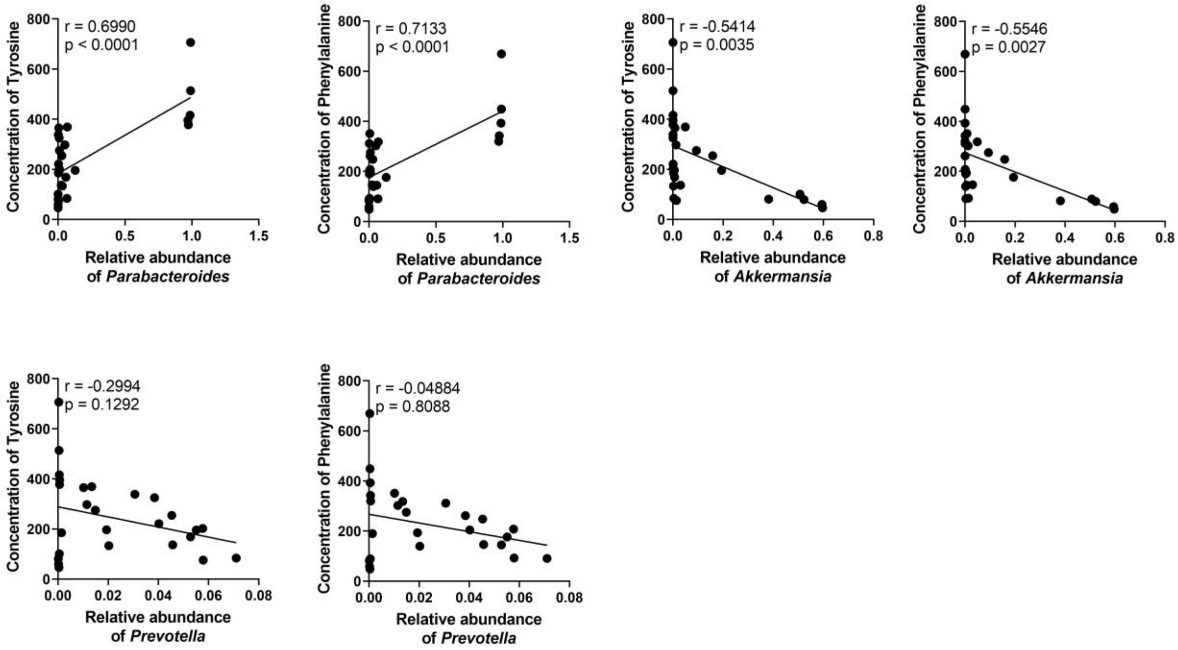

Supplementary Figure S8. The relative gene expression in MLN and Colon in each antibiotic group on day14. The relative expression of gene related to Th1, Th2, Th17, Treg, and pro-inflammation in MLN and Colon (*P < 0.05, **P < 0.01; Mann-Whitney U test and spearman's correlation, CON, control; AVNM, mixture of ampicillin, vancomycin, neomycin and metronidazole; AMP, ampicillin; VAN, vancomycin; MET, metronidazole).

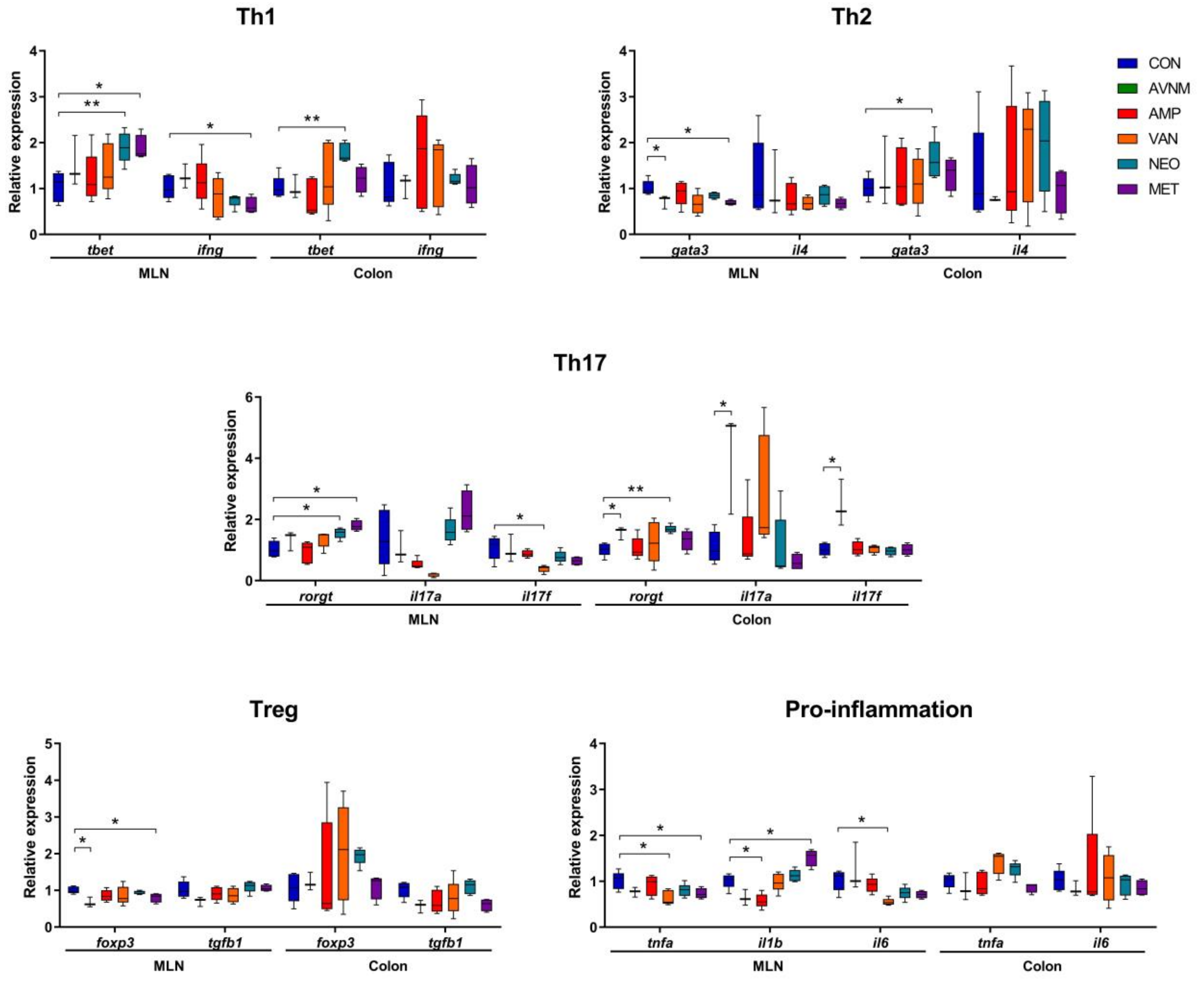

Supplement: Supplementary file 1 [file antibiotics-11-01762-s001.zip › antibiotics-2033874-supplementary/Supplementary files/Supplementary figures/supplementary figures.pdf]
