# Supplementary figures and images for: Distinct Changes in Microbiota-Mediated Intestinal Metabolites and Immune Responses Induced by Different Antibiotics
Source: Antibiotics (Basel). 2022 Dec 6;11(12):1762. doi: 10.3390/antibiotics11121762 (PMC9774394; doi:10.3390/antibiotics11121762)

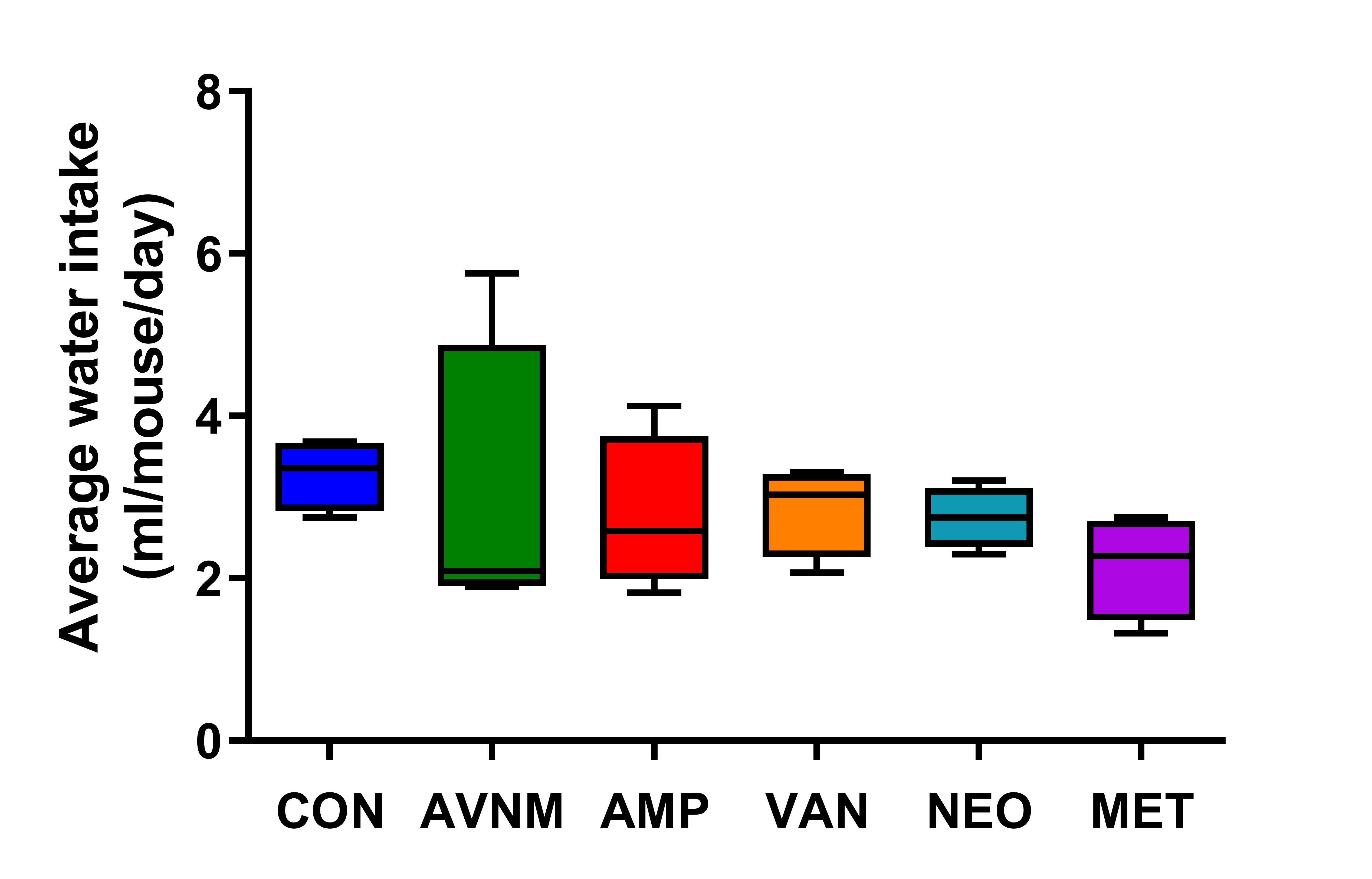

Supplement: Supplementary file 1 [file antibiotics-11-01762-s001.zip › antibiotics-2033874-supplementary/Supplementary files/Supplementary figures/Supplementary Figure S1.jpg]

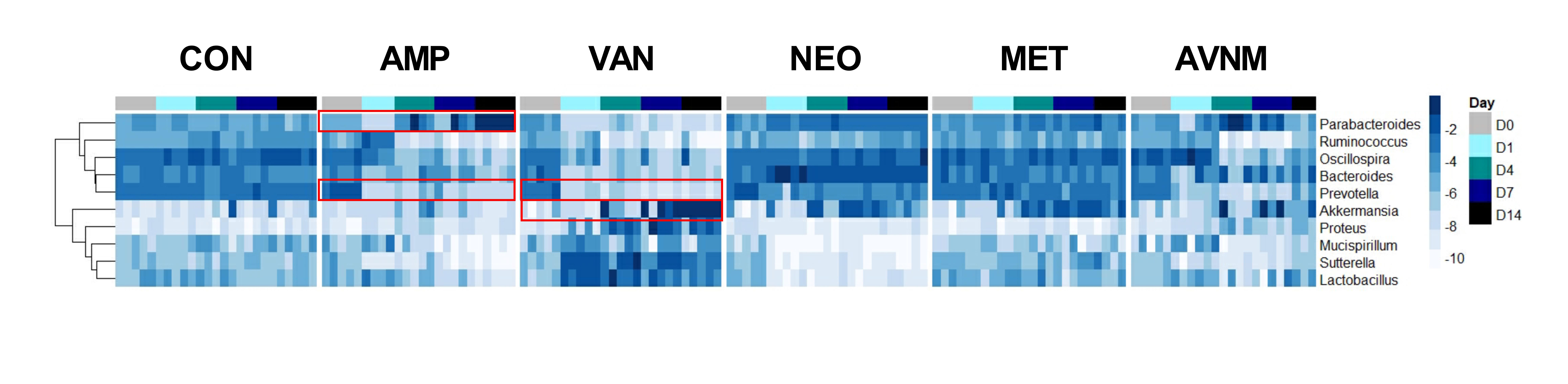

Supplement: Supplementary file 1 [file antibiotics-11-01762-s001.zip › antibiotics-2033874-supplementary/Supplementary files/Supplementary figures/Supplementary Figure S4.jpg]

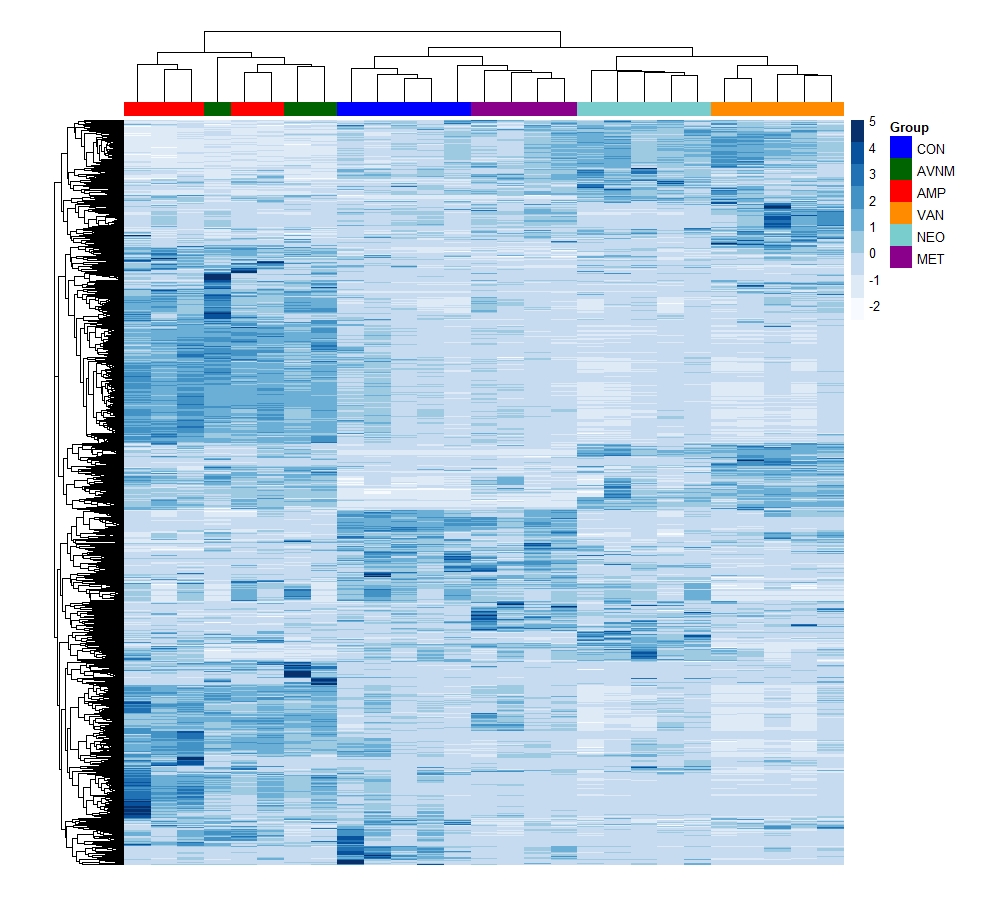

Supplement: Supplementary file 1 [file antibiotics-11-01762-s001.zip › antibiotics-2033874-supplementary/Supplementary files/Supplementary figures/Supplementary Figure S5.jpeg]
